# Intimate partner homicide: victims and the dynamics of victim – perpetrator relationship

**DOI:** 10.1080/07853890.2021.1896181

**Published:** 2021-09-28

**Authors:** Andreia Matias, Mariana Gonçalves, Marlene Matos, Cristina Soeiro

**Affiliations:** a School of Psychology, Universidade do Minho, Braga, Portugal; b Universidade do Minho, Braga, Portugal; d Instituto Universitário Egas Moniz (IUEM), Egas Moniz Cooperativa de Ensino Superior, Caparica, Portugal

## Abstract

**Introduction:**

Intimate partner homicide is considered one of the most extreme forms of interpersonal violence, where one in seven homicides is perpetrated by an actual or former intimate partner. The aim of this study is to analyse the characteristics of the intimate partner homicide (IPH) victims as well as the dynamics of victim - perpetrator relationship.

**Materials and methods:**

The data used in this study were obtained through the analysis of IPH criminal cases on Portuguese courts, that were finalised and handed down and/or sentenced between 2010 and 2015. The relevant authorizations were obtained from several courts, after the approval of this project by the Ethics Committee of Minho University —Subcommittee on Ethics for Social and Human Sciences (SECSH 060/2016). The criminal cases were consulted in several courts at national level – Lisboa and Vale do Tejo (*n* = 53), Vila Real (*n* = 5), Braga (*n* = 4), Vila do Conde (*n* = 3), Aveiro (*n* = 3), Ponta Delgada (*n* = 3), Póvoa do Varzim (*n* = 2), Viana do Castelo (*n* = 1) and Maia (*n* = 1). Our sample was composed by 75 cases, in which 63 (84%) IPH were perpetrated by men and 12 (16%) by women. From those, 19 were homicides-suicides (25%) and 5 homicides-suicide attempts (7%).

**Results:**

IPH victims were aged between 30 and 50 years, mostly were professionally active (*n* = 40; 63%) and from undifferentiated profesisons (*n* = 43; 74%). Most of them did not present substance abuse (*n =* 61; 81%) or psychiatric history (*n* = 60; 80%). Only in 31% (*n* = 23) there was records danger perception. Regarding the relationship between the IPH perpetrator and the victims, it had an average duration of 13.4 years, with a difference of ages of 6.7 months. There were in total 66 children involved in these cases. The relationships were mostly current (*n* = 52; 69%) and formal (*n* = 68; 91%). Of all the analysed cases, although the majority (72%) presented previous history of intimate partner violence (IPV), there was a relevant proportion of cases (28%) without any reported or registered IPV. In 79% (*n* = 59) of cases, the men were the primary aggressors (five women and one man perpetrated IPH in sequence of an history of previous victimization). In 21% (*n* = 16) of cases the reported IPV was bidirectional. Regarding the types of IPV, in 91% (*n* = 68) of the cases registered the presence of previous psychological violence, 61% (*n* = 46) physical violence, 46% (*n* = 35) stalking and 44% (*n* = 33) control behaviours. In 83% (*n* = 62) there were co-occurrence of different types of violence. In 39% (*n* = 29) of the cases with IPV records there were only registers of psychological violence and/or stalking and/or control behaviours.

**Discussion and conclusions:**

These results obtained allowed to conclude that intimate homicide victims don't have a stereotyped profile. Also, these data demonstrate that the victims do not have characteristics that allow them to anticipate their vulnerability. Although most cases of IPH occurred mainly as the culmination of a repeatedly history of prior violence and in situations in which physical violence was a common feature, there is a strong occurrence of psychological violence in IPH. Therefore, the results obtained allow us to identify some essential areas for prevention and intervention. The practical implications of the results are discussed in order to improve the professional practices of the various systems, Integrated in the technical and social systems that must intervene in IPV context.

